# An entropy-based approach to the study of human mobility and behavior in private homes

**DOI:** 10.1371/journal.pone.0243503

**Published:** 2020-12-10

**Authors:** Yan Wang, Ali Yalcin, Carla VandeWeerd

**Affiliations:** 1 Citibank, Tampa, Florida, United States of America; 2 Department of Industrial and Management Systems Engineering, The University of South Florida, Tampa, Florida, United States of America; 3 Department of Health Outcomes and Biomedical Informatics, College of Medicine, The University of Florida, Gainesville, Florida, United States of America; Peking University Shenzhen Graduate School, CHINA

## Abstract

Understanding human mobility in outdoor environments is critical for many applications including traffic modeling, urban planning, and epidemic modeling. Using data collected from mobile devices, researchers have studied human mobility in outdoor environments and found that human mobility is highly regular and predictable. In this study, we focus on human mobility in private homes. Understanding this type of human mobility is essential as smart-homes and their assistive applications become ubiquitous. We model the movement of a resident using ambient motion sensor data and construct a chronological symbol sequence that represents the resident’s movement trajectory. Entropy rate is used to quantify the regularity of the resident’s mobility patterns, and an upper bound of predictability is estimated. However, the presence of visitors and malfunctioning sensors result in data that is not representative of the resident’s mobility patterns. We apply a change-point detection algorithm based on penalized contrast function to detect these changes, and to identify the time periods when the data do not completely reflect the resident’s activities. Experimental results using the data collected from 10 private homes over periods of 178 to 713 days show that human mobility at home is also highly predictable in the range of 70% independent of variations in floor plans and individual daily routines.

## Introduction

Human mobility is the movement of human beings in space and time and may pertain to an individual or a population [1]. Human mobility occurs in varying distance scales ranging from movement by foot within an indoor environment such as homes or buildings to long-distance travel by different modes of transport using cars, buses, and trains in outdoor environments. In recent decades, the pervasion of mobile devices has enabled the collection of large-scale geolocation information related to outdoor human movement facilitating research aimed at gaining a deeper understanding of human mobility. Studies based on ubiquitous data such as call detail records (CDRs) [2,3], GPS logs [4–7], WLAN logs [8], and transportation smart card records [9,10] have shown that human mobility is not completely random but potentially regular and predictable. Understanding human mobility benefits applications including but not limited to urban planning [11,12], epidemic models [13,14], and disaster response [15,16].

In indoor environments, a growing number of context-aware smart home applications including automation [17,18], energy management [19–22], abnormal situation diagnoses [23–26], reminder assistance [27,28] and healthy lifestyle promotion [29] characterized by their ability to be sensitive to occupants’ location, movement, and activity are emerging. Smart homes are increasingly seen as facilitating innovative and supportive environments that provide intelligent services to enable the healthy, safe, and independent aging plan desired by older adults [30,31]. Domestically, programs such as the MAVHome at the University of Texas Arlington [18], the Aware Home at the Georgia Institute of Technology [32], and the Gator Tech Smart House at the University of Florida [33] have historically served as single-home-test-bed style environments. Internationally, the U-Health smart home project at POSTECH [34–36] integrates information from small-sized medical body sensors [37] with other ambient sensors to assist older adults in their homes. Other programs including the Place Lab at the Massachusetts Institute of Technology [38], the Tiger Place project at the University of Missouri-Columbia [39], the CASAS Smart Homes project at Washington State University [40], the ORCATECH project of the Oregon Health and Science University [41], and HomeSense project at the University of South Florida [42] represent multi-unit smart home projects that are testing a variety of devices as a means to impact health and well-being across varying program targets.

The study of human mobility in indoor environments based on ambient sensor data differs from the study of outdoor mobility based on geolocation information in the following five distinct ways.

1. *Data collection infrastructure*: In outdoor environments, mobility information is collected through common infrastructures such as mobile communication networks, GPS satellites, Wi-Fi access points, etc. While in indoor environments such as smart homes, the sensor layouts used to collect information differ from house to house due to different floor plans, sensor density and types, and occupant’s preferences. Furthermore, ambient sensors are more prone to temporary outages due to power and usage-related issues resulting in intermittent loss of data.
2. *Data generating frequency*: In outdoor environments, data are collected when mobile devices are activated (making a call, accessing some location-related services, or connecting to a Wi-Fi access point), and therefore data generation frequency is sparser than that of ambient sensor networks where sensors are triggered passively without any intent by humans.
3. *Data ambiguity*: Mobile devices have unique identifiers linking them to a distinct moving object. On the other hand, data from simple ambient sensors cannot identify one distinct moving object from another. Therefore, visitors and residents in the home would generate a different mobility pattern than only the residents of the home.
4. *Distinct location limits*: In outdoor environments, distinct locations humans can visit are essentially unconstrained. However, in smart home environments, the number of distinct locations is fixed and determined by the installed motion sensors.
5. *The time period for trajectory construction*: In outdoor environments, an individual’s movement over multiple days is modeled as a stationary stochastic process. Typically months of data are needed to capture all visited locations and a single sequence of movements is constructed for each individual in a large population. On the other hand, in smart home environments, a resident repeats routine behaviors on a daily basis. The data collected by ambient sensors facilitates the construction of multiple trajectories for different time periods and enables the study of the changes in human mobility over time.

The design and evaluation of context-aware smart home applications providing adaptive intelligent services for its residents must consider the regularity and predictability of human mobility and behavior at home. The only work we have come across which studies the regularity and predictability of human mobility at home is [43]. In this work, mobility is defined as the number of times an individual moves between different rooms in their home within a specified period of time without explicitly considering location information. The results indicate that while a common model across individuals is absent, a high degree of regularity and predictability of human mobility exists when contextual information e.g. walking speed, age, weather, socioeconomic status, etc. about individuals is taken into consideration. The authors conclude that in-home mobility is also highly stereotyped, albeit in a different way than outdoor mobility, and may have applications in predicting individual human health and functional status by detecting adverse events or trends, and in conducting more meaningful clinical trials.

In this paper, we study human mobility in homes outfitted with ambient sensors. Our objective is to quantify the regularity and predictability of human mobility in private homes. We model an individual’s mobility as a stationary stochastic process and construct trajectories of the occupant using sequences of chronologically visited locations in the home based on the data from ambient motion sensors. The entropy rate of the mobility is estimated from the sequences and represents a quantitative measure of the regularity and the limit of predictability of mobility is estimated using the estimated entropy rate.

The ambiguity associated with the mobility data collected from private homes and the unreliability in the data collection infrastructure introduce significant intermittent deviations to the assumed stationary stochastic process. To capture this unknown number of deviations, we model the time series of daily entropy rate as piecewise constant and estimate these change-points using a change-point detection algorithm. [44,45] provide comprehensive reviews of methods for change-point estimation in sequential data considering variations in model assumptions. A penalized least-square change-point estimator based on the Schwarz’s criterion [46] is introduced in [47] to estimate the unknown number of change-points. In this method, the unknown number of change-points is estimated by minimizing the sum of squares of the residuals combined with a penalty on the number of change-points. It is shown that this least-square estimator is a consistent estimator of the number of change-points under the assumption that the random variables are independent and normally distributed. [48,49] expanded this work to a general context where the variables are not necessarily independent. [50] proposed to estimate the unknown change-points by minimizing a penalized contrast function which converges to the true values with probability. The latter has been used widely in different applications including but not limited to animal trajectory segmentation [51], EEG segmentation [50], CGH data analysis [52], and offset detection in GPS data [53]. In this study, we apply this method to segment the sequence of daily entropy rates to determine unknown changes in the data collection environments.

## Materials and methods

The theoretical fundamentals of human mobility and the background associated with the study of regularity and predictability of human mobility are introduced below. The notations, definitions, and formulas follow those presented in [54] and [2] where entropy rate has been used to quantify the extent to which an individual’s travel patterns are regular and predictable.

### Human mobility model

Human mobility is modeled as a stationary stochastic process ***X*** = {*X*_*i*_}, where $X_{i}\in X$ represents the random variable of the location at time *t*_*i*_, *i* = 1, 2, …, *n*. In this study, X is the set of all motion sensors installed in a house, and *X*_*i*_ is a unique motion sensor in this set.

A trajectory is a sample path of ***X*** and typically represented as a sequence of time-indexed locations. Let *l*_*i*_ represent the location update at time *t*_*i*_, a trajectory is then defined as a time series of locations *l*_1_, *l*_2_, …, *l*_*n*_ with *t*_1_ < *t*_2_ < … < *t*_*n*_. The duration at location *l*_*i*_ is the time difference between *t*_*i*_ and *t*_*i*+1_. In this mobility model, the set of locations refer to the viewing areas of the motion sensors, and the model captures transitions between the viewing areas of the motion sensors and not the motion within the viewing area of an individual motion sensor.

### Entropy rate

In the study of human mobility, *random entropy*, denoted by *S*^*rand*^, measures the uncertainty of an individual’s next location assuming that this individual’s movement is completely random among *N* possible locations, and is calculated as

$$S^{rand}={\text{log}}_{2}N$$ (1)

If the individual’s movement among *N* possible locations follows a probability distribution *p*(*i*), *i* = 1, 2, …, *N*, the entropy rate of this process is then defined as

$$S^{unc}=-\sum_{i=1}^{N}p(i){\text{log}}_{2}p(i)$$ (2)

and is referred to as the *temporal-uncorrelated entropy*. The third entropy rate is the *real entropy* and is denoted by *S*^*real*^. It considers the frequency of the visited locations and the order in which these locations are visited. It is calculated as

$$S^{real}=-\sum_{T^{'}\in T}P(T^{\prime }){\text{log}}_{2}\left[P(T^{\prime })\right]$$ (3)

where *T* represents the sequence of the visited locations and *T*′ represents a subsequence of *T*.

Theoretically *S*^*real*^ ≤ *S*^*unc*^ ≤ *S*^*rand*^. It is important to emphasize that when the process is completely random, *S*^*rand*^ = *S*^*unc*^ = *S*^*real*^, and when the process is not completely random but includes inherent repetitive patterns, *S*^*real*^ is the smallest among the three entropy rate measures.

Given a sequence of length *n* with *N* distinct symbols in the sequence, the value of *S*^*rand*^ is calculated using (1). To calculate *S*^*unc*^ using (2), we need to estimate the probability distribution from the sequence. The probability of *x*_*i*_, *i* = 1, 2, …, *N* is estimated as $\hat{p}(x_{i})=N_{i}/n$, where *N*_*i*_ is the total number of *x*_*i*_ in the sequence. The real entropy *S*^*real*^ cannot be obtained directly using (3) but can be estimated by entropy rate estimators. We estimate the value of *S*^*real*^ based on the Burrows-Wheeler transform (BWT) estimator which is easy to implement and is shown to be almost-sure convergent for stationary, ergodic random processes [55] characteristic of movement trajectories considered in this work.

### The limit of predictability of human mobility

Let *h*_*n*−1_ = {*X*_1_, *X*_2_, …, *X*_*n*−1_} be an individual’s locations at times *t*_1_ through *t*_n−1_ and *P*(*h*_*n*−1_) be the probability of observing *h*_*n*−1_. Let *π*(*h*_*n*−1_) be the probability that an individual will be at his/her most likely location at time *t*_n_. The predictability of the *n th* location given the historical trajectory *h*_*n*−1_, denoted as Π(*n*), is defined as

$$\Pi (n)\equiv \sum_{h_{n-1}}P(h_{n-1})\pi (h_{n-1})$$ (4)

Π(*n*) can be viewed as the highest accuracy to predict an individual’s *n th* location given the historical trajectory *h*_*n*−1_.

Taking the limit, the overall predictability is defined as the averaged predictability over time:

$$\Pi \equiv {\text{lim}}_{n\to \infty }\frac{1}{n}\sum_{i}^{n}\Pi (i)$$ (5)

The upper bound of predictability Π, denoted as Π^*max*^, is obtained by solving

$$S=-{П}^{max}{log}_{2}({П}^{max})-(1-{П}^{max}){log}_{2}(1-{П}^{max})+(1-{П}^{max}){log}_{2}(N-1)$$ (6)

where *S* is the entropy rate and *N* is the number of distinct symbols in the process. Π^*max*^ can be treated as the theoretical highest accuracy that a best designed predictive algorithm can achieve for the next location prediction problem [2].

### Data collection

The data used in this study are collected from HomeSense [42], a smart home project at the University of South Florida that aims to apply ambient intelligence technologies in real living environments to help older adults age in place. All participants of HomeSense live alone without pets in their own homes and are recruited from a 55+ active retirement community. The participants are initially contacted by phone about the potential study. During this call, study aims and requirements are explained to participants, eligibility/enrollment criteria are tentatively verified (for example, the participants are asked to be available for bi-weekly phone interviews designed to collect self-reported information regarding major health and life events, travel, and visitors), and an appointment is set for an in-home visit. During the in-home visit, study goals and needs are recapped and written informed consent is obtained. This study is approved by the University of South Florida Human Research Protection Program. Further details regarding participant recruitment, consent, and participation are outlined in IRB Protocol PRO 00020982.

The sensor array deployed in the homes includes Passive Infrared (PIR) motion sensors, contact sensors, power sensors, water sensors, and environmental sensors that report changes in temperature, luminance, and humidity. PIRs are installed in every room such that their field of vision covers the majority of the space in the room where the occupant is active. In this study of human mobility, only the data from PIR motion sensors are used. Further details regarding the data collection environments can be found in [42].

The dataset includes all participants who were enrolled in HomeSense for at least five months between January 1, 2017 and December 31, 2018, who did not disclose family or friends staying with them long-term, and who did not report significant mental or physical impairments in the bi-weekly assessment. In total 10 homes representing 3812 days of data are initially included in this study. A limitation of this study is that the dataset is collected from participants who are all older adults (age 55+). Further investigation is required to validate the results of the study across other age groups and in increased number of installations.

Subsequently, 21 more days were excluded from the dataset when the participants reported as being on vacation in bi-weekly interviews, and the days which were not reported by the participants but had fewer than 12 motion sensor events in a given day. This threshold was determined using the sensor data from the days where the participants reported as being on vacation. These events correspond to sensor errors and visitors who may have come to check on the house and are not representative of the participants’ typical activities.

### Data preparation

Daily motion trajectories are constructed based on the ON events from PIRs that observe a resident’s movement within the house. An ON event is reported by a motion sensor when a movement is detected in the field of the motion sensor’s view, and a sequence of chronological ON events represents the movement history. The sequence of ON events is transferred to a symbol sequence by replacing each of them by the symbol representation, for example, the sensor identity, to uniquely represent the motion sensor that reports an ON event, and thus we construct a symbol sequence representation of movement trajectory of the resident.

Using the daily motion trajectories and the BWT entropy estimator, we estimate the true daily entropy rate defined in Eq (3) and construct a sequence of daily entropy rates for each home to describe the resident’s mobility over time. Similarly, we also calculate the limit of predictability for each day using (6).

We define outliers as data points for which the estimated daily entropy rates are outside of the [*Q*_1_ − 1.5 * *IQR*, *Q*_3_ + 1.5 * *IQR*] range where *Q*_1_ and *Q*_3_ are the lower and upper quantile of the dataset respectively, and *IQR* = *Q*_3_ − *Q*_1_. Only outliers that do not have another outlier within (±3 days) are removed from the dataset to ensure that temporary shifts are not removed from the dataset. Using this method, we exclude 19 data points reducing the dataset size to 3772 for all houses.

Table 1 summarizes the resulting dataset size for each house, the minimum and the maximum number of unique symbols in the daily trajectories, the minimum, the maximum, and the average length of the daily trajectories. The value of the maximum number of unique symbols denoted as *N*_*max*_, varies between 8 and 12 as a consequence of the different sensor layouts in private homes. For houses with the same *N*_*max*_, the average length of daily trajectories also varies from house to house. For example, the average length of the daily trajectory of House 13 (203) is almost twice as that of House 8 (112) while both of them have *N*_*max*_ = 10, implying that the average movement level of the participant in House 13 is higher than the participant in House 8.

**Table 1:** Summary of the datasets for each house.

House	Size of dataset	*N*_*min*_	*N*_*max*_	Minimum trajectory length	Maximum trajectory length	Averaged trajectory length
8	687	4	10	21	395	112
13	713	4	10	23	545	203
14	178	3	8	28	181	82
27	674	3	8	19	264	96
28	495	6	11	17	542	192
51	178	5	10	15	286	131
53	210	5	9	31	197	92
54	220	6	12	50	492	212
55	208	4	10	37	368	168
56	209	5	10	38	529	173

### Change-point detection algorithm

As discussed in the Introduction section, changes in the data collection infrastructure such as addition or removal of sensors, temporary sensor malfunction which may last days or even weeks, and the presence of long-term visitors significantly alter the patterns in the motion sensor data from the residence and the regularity and predictability of the resident’s mobility estimated based on it. While such events are unavoidable during longitudinal data collection in private homes, identification and exclusion of such periods of time when the collected data is not truly representative of the resident’s normal daily activities will result in a more accurate and representative estimation of the regularity and predictability of the resident’s mobility. To accomplish this, we apply a change-point detection algorithm on the sequence of daily entropy rates to identify segments of time where the sensor data may not be representative of the resident’s normal activity patterns.

Denoting the sequence of daily entropy rate as ***s*** = (*s*_1_, *s*_2_, …, *s*_*n*_) where *n* is the number of days, we model this sequence of daily entropy as piecewise constant [56]

$$s_{j}=\mu_{k}+\varepsilon_{j},0<\tau_{k-1}<j\leq \tau_{k}<n,1\leq k<K$$ (7)

where *K* is the total number of segments, ***τ*** = (*τ*_1_, *τ*_2_, …, *τ*_*K*−1_) with 0 < *τ*_1_ < *τ*_2_ < … < *τ*_*K*−1_ < *n* is the sequence of change-points, *μ*_*k*_ represents the mean of daily entropy in segment *k* which is different for consecutive segments, and *ε*_*j*_ is the error item with a zero mean and a constant variance *σ*^2^.

Estimating change-points where the true number of change-points is unknown can be treated as a model selection problem where the optimal segmentation solution is obtained by minimizing a penalized contrast function.

J(τ,s)+β*pen(τ) (8)

where *J*(***τ***, ***s***) is the contrast function used to measure the contrast between the segmentation marked by ***τ*** and the sequence ***s***, *pen*(***τ***) is the penalty term which increases as the number of change-points increases, and *β* is the penalization parameter or tune parameter that adjusts the minimization of *J*(***τ***, ***s***) and the minimization of *pen*(***τ***).

As suggested in [50], we use

$$J(\tau ,s)=\frac{1}{n}\sum_{k=1}^{K}\sum_{i=\tau_{k-1}+1}^{\tau_{k}}{\left(s_{i}-{\overset{-}{s}}_{\tau_{k-1}+1:\tau_{k}}\right)}^{2}$$ (9)

as the contrast function for the detection of abrupt changes in the mean of the sequential data where ${\overset{-}{s}}_{\tau_{k-1}+1:\tau_{k}}=\frac{1}{n}\sum_{i=\tau_{k-1}+1}^{\tau_{k}}s_{i}$, i.e., the estimate of the mean of data in segment *k*, 1 ≤ *k* ≤ *K*; for the penalty function, we use *pen*(***τ***) = *K*, the number of segments.

When the number of true segments *K* is known, the best estimate of ***τ*** denoted as ${\hat{\tau }}_{K}$ is the sequence of change-points that minimizes the contrast function *J*(***τ***, ***s***). When *K* is unknown, given an upper bound of *K* denoted as *K*_*max*_, we can calculate ${\hat{\tau }}_{K}$ that minimizes the contrast function for all *K*, *K* = 1 … *K*_*max*_. By definition, the best choice of *K*, denoted $\hat{K}$ among these *K*_*max*_ choices is the one that minimizes the summation of the contrast function and the penalty terms *β* * *pen*(***τ***). We determine the best choice of *K* using the automatic procedure described in [50].

After determining the number of change-points $\hat{K}$ and its corresponding segmentation ${\hat{\tau }}_{1},\ldots ,{\hat{\tau }}_{\hat{K}-1}$, we estimate the mean and variance of the daily entropy in each segment using

$${\hat{\mu }}_{k}=\frac{1}{{\hat{\tau }}_{k}-{\hat{\tau }}_{k-1}}\sum_{j={\hat{\tau }}_{k-1}+1}^{{\hat{\tau }}_{k}}s_{j},{\hat{\tau }}_{k-1}<j\leq {\hat{\tau }}_{k},1\leq k\leq \hat{K}$$ (10)

$${\hat{\varepsilon }}_{j}=s_{j}-{\hat{\mu }}_{k},{\hat{\tau }}_{k-1}<j\leq {\hat{\tau }}_{k},1\leq k\leq \hat{K}$$ (11)

### Parameter setting in the change-point detection algorithm

Two parameters are required for the change-point detection algorithm; the minimum number of points in a segment *L*_*min*_, and an upper bound of the number of segments *K*_*max*_. In our experiment, we use *L*_*min*_ = 1 to ensure the detection of all possible change-points. For *K*_*max*_, a value of two to four times the expected number of segments is suggested to give the algorithm some room to work but to avoid overestimating the number of segments [51,57,58]. In this study, the number of changes in the data collection environment and the sensor system, e.g. visitors, sensor system failures, tends to increase as the data collection time period increases. Therefore, longer time periods are more likely to have more change-points. In our experiments, we use the number of weeks contained in the sequential data as the value of *K*_*max*_.

### Validation of change-points

We validate the results of the change-point detection algorithm by checking whether the date of a change-point can be corroborated with the information from three sources; namely the bi-weekly assessments, the maintenance logs, and device battery information collected from the sensor network. We only consider information dated within two days of a change-point as corroborating evidence.

Bi-weekly assessments include information regarding long-term visitors from the participants. In most cases, this information pertains only to visitors who stay with the participant multiple days/weeks, and in many cases the start and end dates of the visit are approximations.

Maintenance logs are used to record the team’s maintenance work on the sensor network. Logged maintenance activities include replacement of malfunctioning sensors, repositioning sensors, adding and removing sensors, and replacing batteries all of which impact the observed data. In most cases, to minimize the interruptions to the participants’ daily lives, multiple maintenance operations, such as adjusting sensors and replacing batteries, are completed during the same visit.

The third source of information is the data collected from individual devices regarding their battery levels. We use this information to schedule maintenance visits to replace batteries before they are completely drained. If battery replacement is not completed in time and the batteries are completely drained, the device stops reporting data. In such cases, the observed data from the residence, and subsequently the estimates of entropy rates, are not representative of the resident’s normal activity patterns.

The validation process entails using the corroborating information from the three sources for the start date of each segment to classify it into one of five categories: (1) *Single-occupant* when the sensor network is completely functional and system is observing only the participant’s activities; (2) S*ystem-change* when additional motion sensors are added to the system creating a new mode of ‘Single-occupant’; (3) *System-malfunction* when one or more motion sensors malfunction and fail to report data including drained batteries; (4) *Multiple-occupant* when long-term visitors are present, and (5) *Unknown* when we were unable to find corroborating information from bi-weekly assessments or maintenance logs to describe the segment. The segments categorized as *Single-occupant* and S*ystem-change*, denoted as ‘Single-occupant (1)’ and ‘Single-occupant (2)’ respectively, are considered to contain data that is representative of the resident’s normal daily activities and used for further data analysis.

### Illustrative example

**Fig 1:**
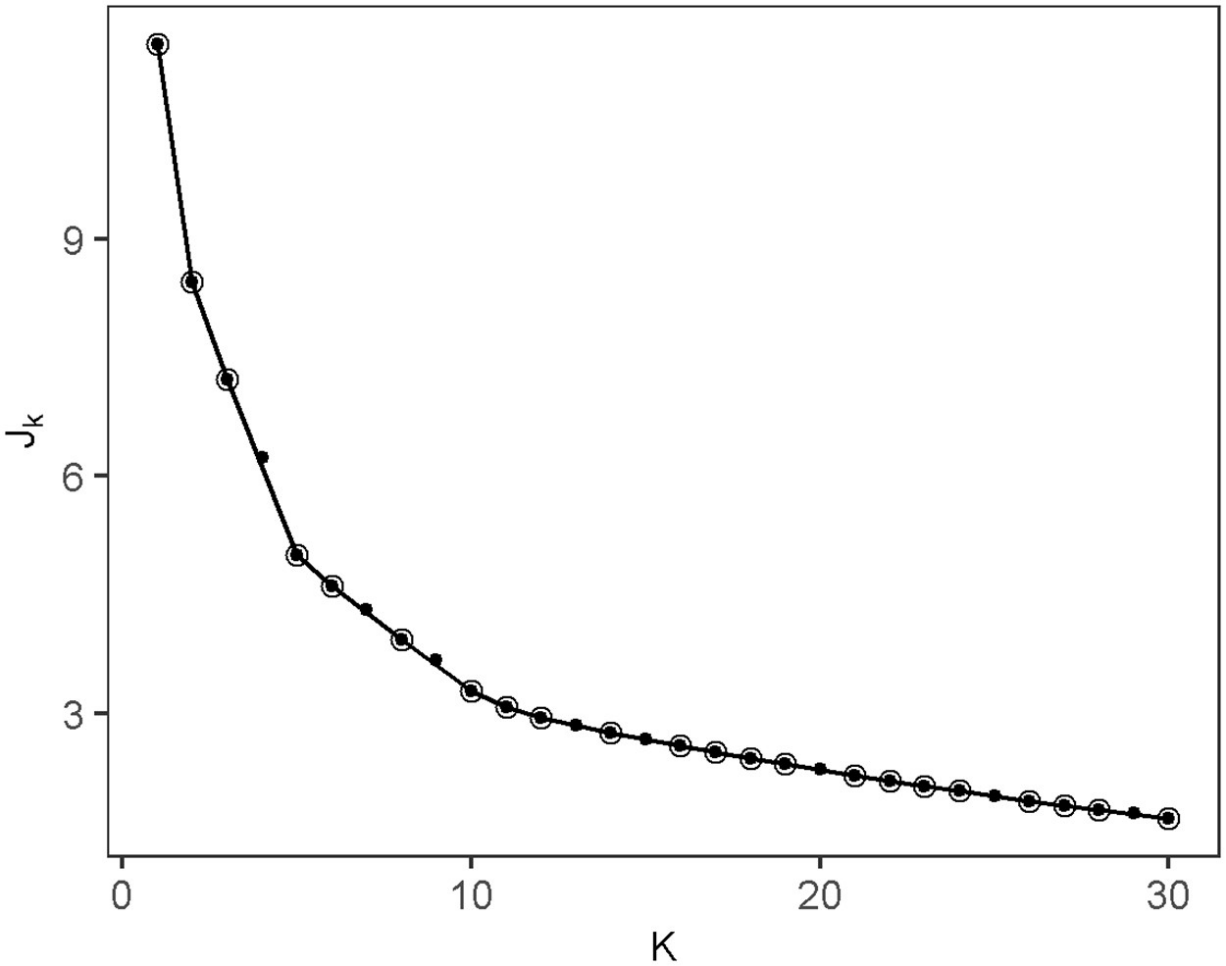
The value of the contrast function *J*_*K*_ for 1 ≤ *K* ≤ *K*_*max*_ = 30 for House 55. Circles indicate the convex hull points of (*K*, *J*_*K*_).

We use House 55 as an example to illustrate the application of the change-point-detection algorithm on the sequence of daily entropy rates, and the validation of the detected change-points. The dataset for House 55 has 30 weeks of data. Thus we set the algorithm parameters as *K*_*max*_ = 30, *L*_*min*_ = 1. Fig 1 shows the value of the contrast function *J*_*K*_ for 1 ≤ *K* ≤ 30. Using the procedure in [50], the optimal number of segments is determined as *K* = 5. Table 2 shows the segments and the results of the change-point validation process used to categorize each of the segments.

**Table 2:** Five segments obtained by the change-point detection algorithm in House 55.

Segment	Number of data points	Date start	${\overset{-}{S}}^{real}$ (SD)	${\overset{-}{\Pi }}^{real}$ (SD)	Interpretation of the start date	Segment type
1	31	2018-06-05	1.48 (0.16)	0.74 (0.036)	Not applicable	Single-occupant (1)
2	33	2018-07-06	1.18 (0.14)	0.80 (0.026)	Unknown	Unknown
3	102	2018-08-08	1.46 (0.15)	0.75 (0.032)	Replace a malfunction sensor	Single-occupant (2)
4	29	2018-11-19	1.82 (0.20)	0.67 (0.047)	Visitor activity	Multiple-occupant
5	13	2018-12-19	1.45 (0.10)	0.74 (0.030)	Unknown	Unknown

**Fig 2:**
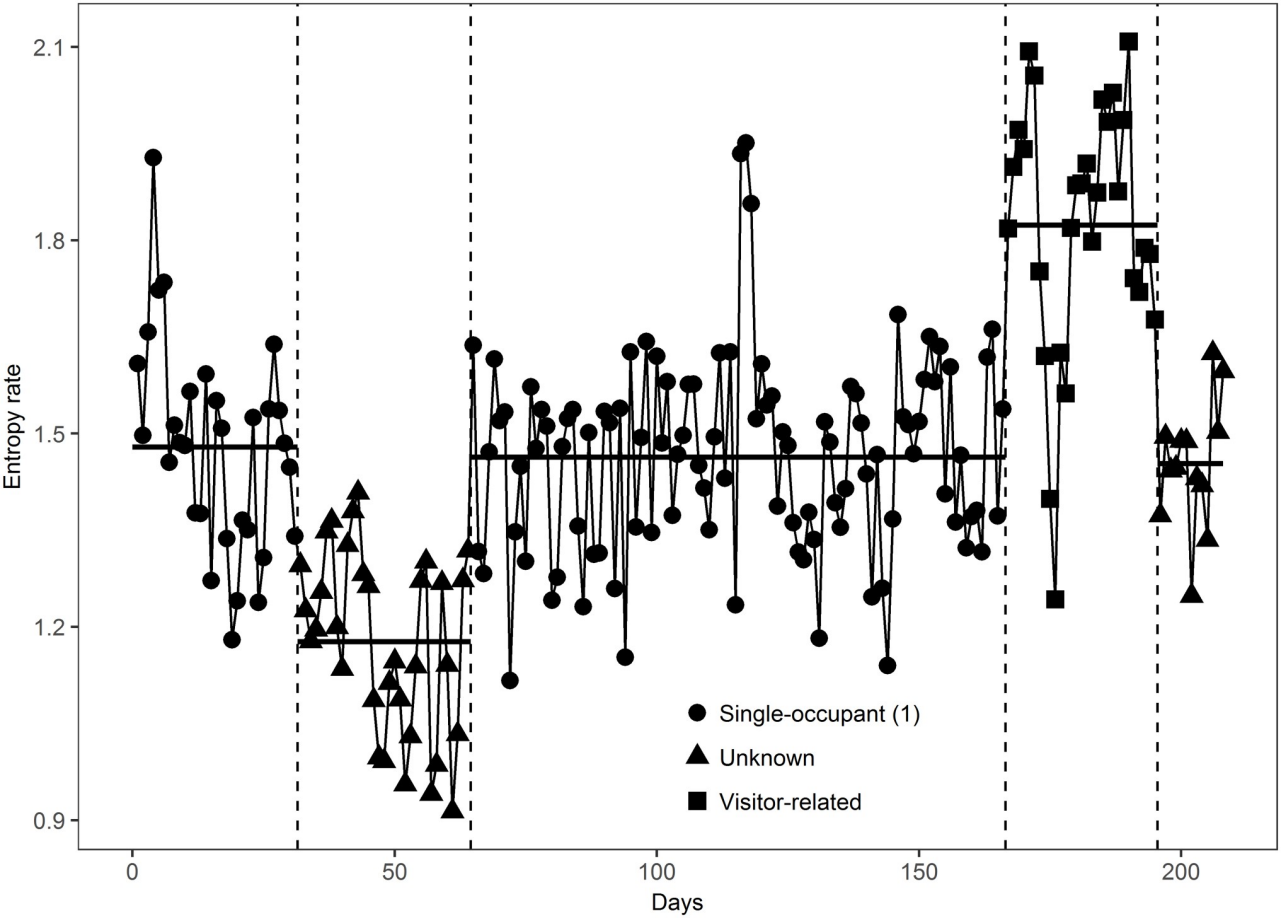
The daily entropy rates in five segments for House 55. The black horizontal lines in the graph show the sample means of the daily entropy rate for each segment, and the vertical dashed lines indicate the location of four change-points.

The first segment in Table 2 which covers the dates between June 5, 2018 and July 6, 2018 is categorized as ‘Single-occupant (1)’ based on our best judgment of the system state at that date using the totality of information from bi-weekly assessments and maintenance logs. This categorization is not based on the change-point detection algorithm as the starting point for this segment is the starting date of the dataset. For the second segment, there is no corroborating information for the change-point found at its start date, and thus it is categorized as ‘Unknown’. The start date of the third segment August 8, 2018 coincides with a maintenance visit where corrections were made to sensors that were not reporting data and therefore this segment is categorized as ‘Single-occupant (2)’. The start date of the fourth segment coincides with visitor arrival and the segment is categorized as ‘Multiple-occupant’. The start date of the fifth segment could not be corroborated with any record in the maintenance logs and bi-weekly assessments and therefore this segment is categorized as ‘Unknown’. Fig 2 illustrates the five segments of the sequence of daily entropy rates.

This systematic approach to categorizing segments revealed interesting points of change, where the start of a number of ‘Unknown’ segments related to changes in the resident’s life patterns and marked behavioral changes. For example, compared with the fourth segment, sensor events reported by the motion sensors installed in the master bedroom and master bathroom were absent in early mornings starting on December 18, 2018. While this change in the motion sensor events could not be captured by the bi-weekly phone interviews or the maintenance logs, it is caused by the changes in the occupant’s behaviors which explain the change characterizing the fifth segment.

We observe in Table 2 that the mean of the entropy rate and the mean of the limit of predictability changes in successive segments. The p-values of the Welch’s t-test [59] for pairwise comparisons of the segments in Table 2 are shown in Table 3. The pairwise comparisons between the mean daily entropy rates and predictability of ‘Single-occupant’ segments are significantly different at the 0.01 level than those of ‘System-malfunction’ and ‘Multiple-occupant’ categories, and the results are mixed in the comparisons with the ‘Unknown’ category.

**Table 3:** The p-values of the t-tests of the daily entropy rate (predictability) for pairs of different types of segments in House 55.

	**Segment 2 (Unknown)**	**Segment 3 (Single-occupant (2))**	**Segment (Multiple-occupant)**	**Segment 5 (Unknown)**
**Segment 1 (Single-occupant (1))**	7.96e-11 (1.73e-08)	0.63 (0.57)	1.51e-09 (1.00e-08)	0.53 (0.42)
**Segment 2 (Unknown)**		3.23e-14 (1.85e-12)	1.81e-19 (3.17e-16)	2.26e-08 (3.54e-06)
**Segment 3 (Single-occupant (2))**			6.75e-11 (5.38e-10)	0.75 (0.17)
**Segment 4 (Multiple-occupant)**				9.65e-10 (5.34e-06)

## Results

### Overall entropy rate and limit of predictability

Table 4 shows the sample mean, the range of the random, temporal-uncorrelated, and true daily entropy rates over days, and the corresponding limits of predictability for each house. For the entropy measures, the sample mean of the real entropy ${\overset{-}{S}}^{real}$ is lower than the mean of the temporal-uncorrelated entropy ${\overset{-}{S}}^{unc}$ and the mean of the random entropy ${\overset{-}{S}}^{rand}$, providing evidence that there are inherent repetitive patterns in the daily trajectories of the residents. Similar observations are made for the limit of predictability but with a reverse relationship where the mean of the limit of predictability for the real entropy ${\overset{-}{\Pi }}^{real}$ is the highest. Overall, the sample mean of the real entropy is between 0.48 and 2.36 with a mean of 1.60, and the corresponding limit of predictability is between 54% and 92% with a mean of 72%.

**Table 4:** The sample means and range of entropy rate and the limit of probability.

House	${\bar{S}}^{rand}[S_{min}^{ramd},S_{max}^{ramd}]$	${\bar{S}}^{unc}[S_{min}^{unc},S_{max}^{unc}]$	${\bar{S}}^{real}[S_{min}^{real},S_{max}^{real}]$	${\bar{\prod }}^{rand}[\prod_{min}^{rand},\prod_{max}^{rand}]$	${\bar{\prod }}^{unc}[\prod_{min}^{unc},\prod_{max}^{unc}]$	${\bar{\prod }}^{real}[\prod_{min}^{real},\prod_{max}^{real}]$
8	2.91 [2.00, 3.32]	2.45 [1.86, 2.84]	1.57 [1.14, 2.29]	0.14 [0.10, 0.25]	0.46 [0.33, 0.60]	0.73 [0.54, 0.83]
13	2.92 [2.00, 3.32]	2.65 [1.86, 3.15]	1.82 [1.20, 2.36]	0.14 [0.10, 0.25]	0.37 [0.20, 0.55]	0.67 [0.56, 0.75]
14	2.41 [1.58, 3.00]	2.01 [1.19, 2.64]	1.31 [0.48, 2.01]	0.20 [0.13, 0.33]	0.52 [0.34, 0.73]	0.76 [0.63, 0.92]
27	2.55 [1.58, 3.00]	2.24 [1.28, 2.70]	1.53 [0.70, 2.14]	0.18 [0.13, 0.33]	0.45 [0.26, 0.72]	0.71 [0.59, 0.89]
28	3.15 [2.58, 3.46]	2.59 [2.10, 3.03]	1.71 [1.23, 2.34]	0.12 [0.092, 0.17]	0.46 [0.32, 0.61]	0.71 [0.57, 0.80]
51	3.09 [2.32, 3.32]	2.35 [1.64, 2.73]	1.58 [1.17, 2.00]	0.12 [0.10, 0.20]	0.53 [0.35, 0.70]	0.74 [0.66, 0.82]
53	2.81 [2.32, 3.17]	2.41 [2.05, 2.76]	1.47 [1.14, 1.88]	0.15 [0.11, 0.20]	0.45 [0.31, 0.58]	0.75 [0.67, 0.82]
54	3.18 [2.58, 3.58]	2.40 [1.44, 2.89]	1.66 [1.00, 2.14]	0.11 [0.084, 0.17]	0.53 [0.29, 0.75]	0.73 [0.61, 0.85]
55	2.78 [2.00, 3.32]	2.33 [1.72, 2.76]	1.47 [0.91, 2.11]	0.15 [0.10, 0.25]	0.48 [0.28, 0.62]	0.74 [0.60, 0.84]
56	2.61 [2.32, 3.32]	2.11 [1.68, 2.53]	1.42 [1.05, 2.12]	0.17 [0.10, 0.20]	0.52 [0.38, 0.67]	0.74 [0.62, 0.82]
Overall	2.85 [1.58, 3.58]	2.41 [1.19, 3.15]	1.60 [0.48, 2.36]	0.14 [0.084, 0.33]	0.45 [0.20, 0.75]	0.72 [0.54, 0.92]

### Results from the change-point detection algorithm

The real entropy rate measures the extent to which movement patterns are regular. Changes in the regular movement patterns that are caused by changes in sensor system configuration or the visitors’ activities could introduce changes in the value of the real entropy rate. The results in this subsection pertain to the analysis of the sequence of daily real entropy rate for each house and use the change-point detection algorithm to examine how it changes over time.

Table 5 shows the segments determined by the change-point detection algorithm, and the segment categorizations using the validation process except for House 55 which is previously shown in Table 2. 37 change-points are detected over 10 houses, and 22 out of them are validated by the records of bi-weekly assessment and the maintenance log.

**Table 5:** Segments of the sequence of daily entropy rates over nine houses and the validation results.

House	Segment	Number of data points	Start Date	${\overset{-}{S}}^{real}$ (SD)	Interpretation of start date	Segment type
8	1	22	2017-01-01	1.42 (0.14)	Not applicable	Single-occupant (1)
	2	36	2017-01-24	1.89 (0.15)	Visitors arrived	Multiple-occupant
	3	233	2017-03-01	1.55 (0.14)	Visitors left	Single-occupant (1)
	4	11	2017-11-03	1.82 (0.10)	Visitors arrived	Multiple-occupant
	5	358	2017-11-14	1.54 (0.13)	Visitors left	Single-occupant (1)
	6	27	2018-12-05	1.68 (0.15)	Unknown	Unknown
13	1	222	2017-01-01	1.66 (0.13)	Not applicable	Single-occupant (1)
	2	180	2017-08-15	1.88 (0.12)	Add a new sensor	Single-occupant (2)
	3	13	2018-02-11	2.22 (0.11)	Visitors arrived	Multiple-occupant
	4	298	2018-02-25	1.89 (0.12)	Visitors left	Single-occupant (2)
14	1	38	2017-01-01	1.23 (0.19)	Not applicable	Single-occupant (1)
	2	54	2017-02-08	0.93 (0.18)	Unknown	Unknown
	3	86	2017-04-03	1.59 (0.15)	Adjust sensors	Single-occupant (2)
27	1	21	2017-01-01	1.09 (0.20)	Not applicable	System-malfunction
	2	66	2017-01-25	1.29 (0.16)	Replace battery	Single-occupant (1)
	3	126	2017-04-01	1.44 (0.16)	Unknown	Unknown
	4	126	2017-08-15	1.62 (0.16)	Add two sensors	Single-occupant (2)
	5	57	2017-12-21	1.40 (0.16)	Unknown	Unknown
	6	192	2018-03-13	1.69 (0.14)	Replace battery	Single-occupant (2)
	7	86	2018-09-30	1.58 (0.17)	Unknown	Unknown
28	1	38	2017-07-07	1.56 (0.14)	Not applicable	Single-occupant (1)
	2	114	2017-08-15	1.72 (0.12)	Add a new sensor	Single-occupant (2)
	3	8	2017-12-16	2.01 (0.16)	Visitors Arrival	Multiple-occupant
	4	175	2017-12-30	1.76 (0.13)	Visitors left	Single-occupant (2)
	5	64	2018-07-12	1.62 (0.12)	Unknown	Unknown
	6	6	2018-09-27	2.09 (0.16)	Unknown	Unknown
	7	90	2018-10-03	1.70 (0.12)	Unknown	Unknown
51	1	21	2018-05-14	1.32 (0.11)	Not applicable	System-malfunction
	2	157	2018-06-11	1.62 (0.14)	Replace Sensor	Single-occupant (1)
53	1	60	2018-05-23	1.43 (0.17)	Not applicable	Single-occupant (1)
	2	53	2018-07-23	1.53 (0.12)	Unknown	Unknown
	3	28	2018-09-26	1.33 (0.12)	Drained batteries	System-malfunction
	4	69	2018-10-24	1.51 (0.14)	Replaced batteries	Single-occupant (1)
54	1	37	2018-05-21	1.61 (0.18)	Not applicable	Single-occupant (1)
	2	30	2018-06-27	1.42 (0.17)	Unknown	Unknown
	3	41	2018-07-27	1.64 (0.13)	Unknown	Unknown
	4	16	2018-09-11	1.94 (0.13)	Network Problem	System-malfunction
	5	96	2018-09-27	1.72 (0.13)	Reinstall Sensor	Single-occupant (1)
56	1	66	2018-06-04	1.53 (0.10)	Not applicable	Single-occupant (1)
	2	5	2018-08-10	1.96 (0.12)	Unknown	Unknown
	3	22	2018-08-15	1.51 (0.11)	Unknown	Unknown
	4	116	2018-09-07	1.32 (0.11)	Drained battery	System-malfunction

Table 6 summarizes aggregate statistics by segment type from all homes. Note that around 50% of the segments containing 75% of the days correspond to normal behavior. ‘Multiple-occupant’ and ‘System-malfunction’ type segments correspond to around 20% of the segments and less than 10% of the days. ‘Single-occupant’ type segments are clearly longer containing a significantly higher number of days than those that correspond to visitors and system malfunction. 30% of segments that contain 20% of the days were categorized as ‘Unknown’.

**Table 6:** Aggregate statistics (mean, (standard deviation) [minimum, maximum]) of daily entropy rate and limit of probability of different types of segments over 10 houses.

Type	Single-occupant (1)	Single-occupant (2)	Single-occupant (1) & (2)	Multiple-occupant	System-malfunction	Unknown	Overall
**Num. of segments**	15	7	22	5	5	15	47
**Num. of days**	1595	1171	2766	97	202	707	3772
${\overset{-}{S}}^{real}$ **(SD) [min, max]**	1.55 (0.17) [0.81, 2.10]	1.77 (0.17) [1.16, 2.22]	1.64 (0.20) **[0.81, 2.22]**	1.92 (0.20) [1.24, 2.36]	1.35 (0.22) [0.70, 2.14]	1.49 (0.26) [0.48, 2.34]	1.60 (0.24) **[0.48, 2.36]**
${\overset{-}{\Pi }}^{real}$ **(SD) [min, max]**	0.73 (0.040) [0.56, 0.86]	0.69 (0.039) [0.57, 0.82]	0.71 (0.044) [0.56, 0.86]	0.65 (0.045) [0.54, 0.80]	0.76 (0.044) [0.61, 0.89]	0.73 (0.052) [0.57, 0.92]	0.72 (0.048) [0.54, 0.92]

Another observation related to the results in Table 6 is that the range of daily entropy rate of ‘Single-occupant’ segments [0.81, 2.22] is much narrower than the range of all segments [0.48, 2.36] indicating that those days with uncharacteristically small and large daily entropy rates were not representative of the residents’ normal routines, but were associated with disruptions which involved the presence of visitors or problems with the ambient sensor system.

### Comparison of entropy rates between segment types

We compare the mean of daily entropy rate of different types of segments within each house to see if there are statistically significant differences between entropy rates of these segments. The results of the 99 pairs of comparisons using Welch’s t-test are summarized in Table 7. All ‘Multiple-occupant’ segments have significantly different means from the ‘Single-occupant’ segments, and all nine ‘System-malfunction’ segments have significantly different means from the ‘Single-occupant’ ones. As expected, the comparison of means with ‘Unknown’ segments has mixed results.

**Table 7:** The number of t-test with p-value < 0.01 versus the number of t-test with p-value > = 0.01 for comparing the means of entropy rate in two different types of segments.

	**Single-occupant (2)**	**Multiple-occupant**	**System-malfunction**	**Unknown**
**Single-occupant (1)**	7 vs. 0	**10 vs. 0**	**7 vs. 0**	16 vs. 6
**Single-occupant (2)**		**4 vs. 0**	**2 vs. 0**	11 vs. 2
**Multiple-occupant**				6 vs. 1
**System-malfunction**				8 vs. 0

### Analysis of normal days’ entropy rates

After isolating the ‘Single-occupant’ segment types that capture the routine behavior of the residents, we proceeded to compare entropy rates of these days within and across homes to determine if there are meaningful subgroups or trends. We first compared entropy rates of weekdays with weekends using Welch’s t-test. The sample means of daily entropy rates for weekdays and weekends were 1.64 and 1.63 respectively, showing no significant difference between the means (p-value = 0.26). For this cohort, we did not expect to see a difference as only one participant has a routine work schedule. This participant works 10 hours each day on Wednesday, Thursday, and Friday. Comparison of the entropy rates of the three working days with the non-working days also did not show any significant differences between the means of daily entropy rates (p-value = 0.51).

**Fig 3:**
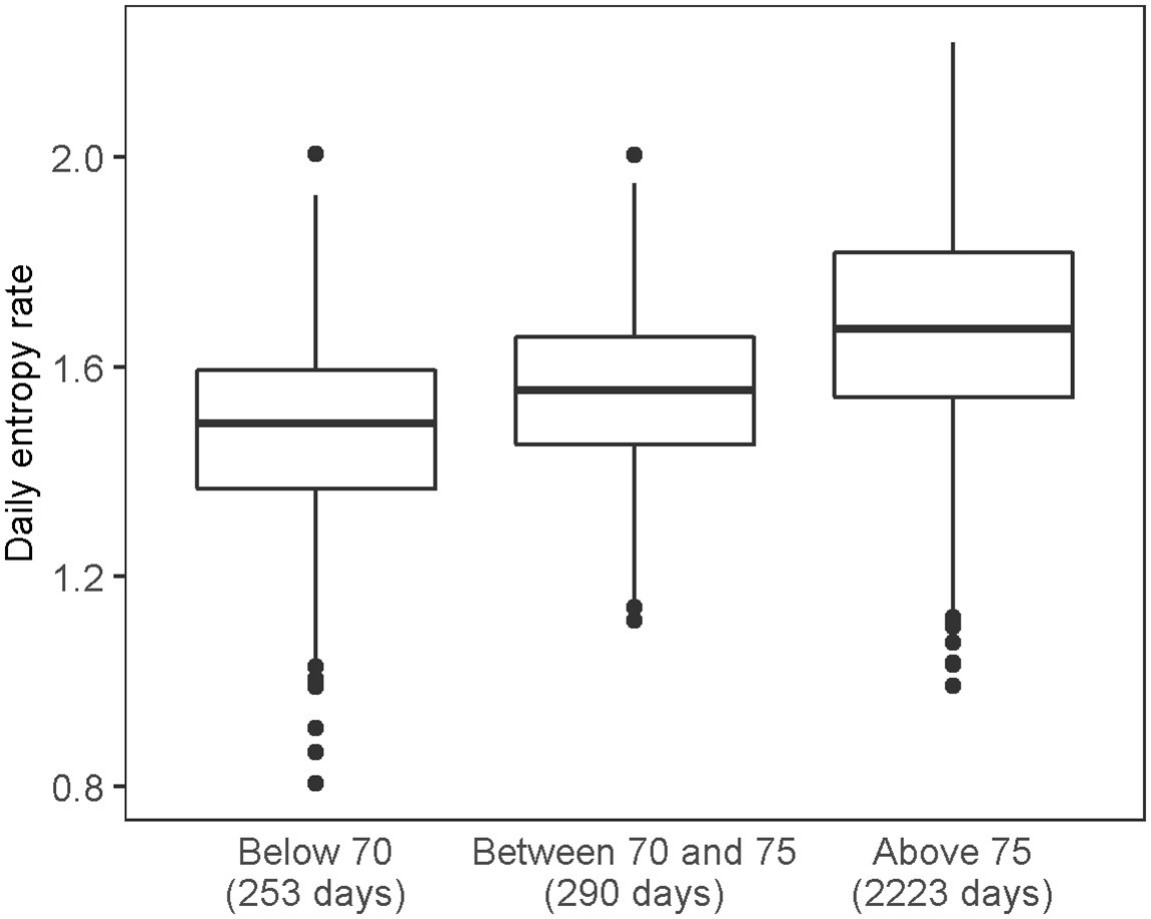
Box plots of the real entropy rates for three age cohorts.

When we studied the daily entropy rates of the participants stratified by age group, we obtained very interesting results. Of the 10 participants, two are below age 70, two are between the ages of 70 and 75, and six are older than 75. The box plots of the entropy rates for these three age groups are shown in Fig 3. The sample means are 1.48, 1.55, and 1.67 respectively and show statistically significant differences (p-value < 0.001) in the daily entropy rates among different age cohorts.

## Discussion

In this paper, we studied human mobility in private homes using data from ambient sensors that observe residents’ movements. We construct daily movement trajectories based on the collected sensor data and use the entropy rate to measure the regularity and predictability of these trajectories. Our analysis shows that the movements of these residents at home are not completely random, but inherently regular and are predictable. The average real entropy for daily trajectories range between 0.81 and 2.22, and their corresponding limit of predictability is between 0.56 and 0.86 (Table 6). On average, about 70% of the time the resident’s next location can be correctly predicted by a theoretically best designed predictive algorithm. The regularity and predictability of the resident’s movements under conditions representative of normal life routines, across different homes with varying floor plans, and for individuals with different lifestyles remained within a very narrow range over long periods of time. This is a very important finding and a unique contribution of this research. To our knowledge, it is the only work of this kind to quantify the predictability of human mobility in private homes and demonstrate its consistency across 10 installations and 3772 days of data.

The data collected from wireless ambient sensor systems in private homes over extended periods of time contains temporary shifts predominantly due to the presence of visitors in the homes and malfunctions in the sensor systems. These factors skew the data collected from the home in the form of missing sensor data in the case of system malfunctions, and additional sensor data not representative of the resident’s movements in the case of visitors. A change-point detection algorithm is used to identify such segments of time and study their influence on the entropy rates of daily trajectories. Results of the change-point detection algorithm shown in Tables 5 and 6 present clear differences between the entropy rates of days that belong to different types of segments.

Using the bi-weekly phone interviews with the participants and maintenance logs to corroborate the change-points from the algorithm, the segments were classified into five categories as ‘Single-occupant’, ‘System-change’, ‘Multiple-occupant’, ‘System-malfunction’, and ‘Unknown’. 75% of the study days corresponded to the normal behavior of the participant without the effects of known artifacts such as visitors and sensor system malfunctions. ‘Multiple-occupant’ and ‘System-malfunction’ type segments corresponded to less than 10% of the days, and 20% of the days were categorized as ‘Unknown’ as the starting change-points could not be validated by the interviews and logs. However, we were able to anecdotally observe behaviors from the rest of the sensor data which could have caused changes in daily entropy rate associated with the behavior of the participant such as changes in sleeping habits which coincided with the start of an unknown period. We note the detection of participants’ behavioral changes using entropy rate as an important future research direction.

‘Single-occupant’ type segments were much longer in duration and contained a significantly higher number of days than those that correspond to visitors and system malfunction. While the average daily entropy rate of the normal days was comparable to the overall average daily entropy rate (1.64 vs 1.60, Table 6), the range of observed daily entropy values of the normal days was significantly narrower. We also observed consistent and statistically significant differences in the means of daily entropies for days categorized as ‘Single-occupant’ vs. ‘System-malfunction’ and ‘Multiple-occupant’ as shown in Table 7. The mean daily entropy rate of visitor days was on average higher than days categorized as ‘Single-occupant’ and ‘System-malfunction’. This is somewhat intuitive as during these days the presence of visitors in the house increased the amount of entropy rate. On the other hand, days during which there were sensor malfunctions where one or more sensors failed to send data, the average daily entropy rate was lower.

After isolating the effect of known causes on the daily entropy rate and focusing on days categorized as ‘Single-occupant’ segments, we proceeded to analyze the data across homes to identify potential patterns. Since our participants are retired older adults, we did not observe any significant differences in daily entropies between weekdays and weekends. Analysis of the daily entropies of the days of the week for one of our participants who works a regular schedule three days a week also did not show significant differences in daily entropy. While this is a very small dataset, it does provide additional evidence that an entropy-based approach is robust to varying lifestyles and routines.

The most interesting results were obtained when analyzing daily entropy rate stratified by age group. We observed statistically significant increases in average daily entropy rate for older cohorts as shown in Fig 3. While our dataset is small based on 10 participants, this is a novel and interesting finding which motivates further study of entropy-based metrics that measure the amount of disorder in stochastic processes as part of an ambient home monitoring system to identify aging-related behavior changes.

Overall, 60% of the change-points detected by the algorithm are validated by the information in the bi-weekly phone interviews with the participants and maintenance and system logs. Since the information from the logs are incomplete, and there were other potential sources of change in the data collected from the private homes such as the changes in the resident’s behavior, we believe this percentage of validation is in fact very promising in terms of further investigating entropy-based metrics as part of a comprehensive activity and overall health monitoring system in more structured and closely monitored experimental designs.

Identification of periods of time which are skewed by factors other than participants’ behaviors is essential for effective monitoring of health and wellness using ambient sensor systems in private homes. In this initial phase we isolated time periods which are not representative of the participant’s behavior, and prepared the data set to detect finer changes in behavior such as sleeping and hygiene habits that could be linked to changes in health and wellness. Detecting and validating these arguably more subtle changes is challenging. From a methodology perspective, [7] introduces an information-theoretic metric “instantaneous entropy” which allows a per time slot view of the entropy rate. This metric is used to quantify changes in the unpredictability of individual mobility and is shown to outperform a single summary entropy rate in detecting abnormal deviations of mobility routines. Information-theoretic metrics are model-free, provide an informative understanding of human mobility, and present great potential to be considered as features to detect behavior changes to facilitate health and wellness monitoring at home.

Another significant research challenge lies in the validation of these methodologies. Traditional approaches such as activity logs and cameras have well-known limitations [42] to document even the most well-defined activities of daily living. Subtle behavior changes that are signs of worsening chronic conditions and other changes in health and wellness are much more difficult to establish and require rethinking traditional validation methods aimed at identifying these changes.
